# The effectiveness of vibration therapy for muscle peak torque and postural control in individuals with anterior cruciate ligament reconstruction: a systematic review and meta-analysis of clinical trials

**DOI:** 10.1186/s10195-021-00589-5

**Published:** 2021-07-14

**Authors:** Nastaran Maghbouli, Mahmoud Khodadost, Saeed Pourhassan

**Affiliations:** 1 Physical Medicine and Rehabilitation Department, Tehran University of Medical Sciences, Tehran, Iran; 2 Department of Epidemiology, School of Public Health, Shahid Beheshti University of Medical Sciences, Tehran, Iran; 3 Department of Epidemiology, School of Public Health, Larestan University of Medical Sciences, Larestan, Iran; 4 Internal Medicine Department, Shariati Hospital, Tehran University of Medical Sciences, 1411713135 Tehran, Iran

**Keywords:** Anterior cruciate ligament reconstruction, Vibration, Torque, Postural balance, Rehabilitation

## Abstract

**Objective:**

This study aimed to review and summarize the existing evidence on the effectiveness of vibration therapy (VT) in comparison with conventional rehabilitation in anterior cruciate ligament (ACL)-reconstructed patients considering muscle peak torque and postural control.

**Methods:**

We searched available online databases for relevant studies published up to February 2020. All randomized clinical trials investigating the effect of VT on quadriceps peak torque, hamstring peak torque, and postural control (closed-eye and open-eye) were included. Overall, 13 clinical trials with a total sample size of 407 participants were included for the meta-analysis. We used the pooled mean difference with random effects model for meta-analyses. We assessed the heterogeneity of the studies using the *I*^2^ and Cochran’s *Q* test. Meta-regression analysis was used to assess the source of heterogeneity.

**Results:**

We found that VT significantly improved hamstring peak torque [weighted mean difference (WMD) 12.67, 95% CI 4.51–20.83] and quadriceps peak torque (WMD 0.11, 95% CI −0.06 to 0.29). However, subgroup analysis showed a significant increase in mentioned muscles’ peak torque in studies employing interventions including both local muscle vibration (LMV) and vibration frequency higher than 100 Hz (WMD 20.84, 95% CI 11.75–29.93). With regard to postural control, we observed a significant improvement only in open-eye mediolateral postural control (WMD 0.26, 95% CI −1.26 to 1.77).

**Conclusion:**

This study suggests that VT, especially LMV type with vibration frequency higher than 100 Hz, can be effective in rehabilitation of ACL-reconstructed patients. Although improvement in the peak torque of hamstring and quadriceps muscles was seen, there was no significant improvement in postural control, especially closed-eye, in comparison with conventional rehabilitation.

**Level of evidence:**

1.

**Highlights:**

Vibration therapy can increase hamstring peak torque in individuals with ACL reconstruction.

Local muscle vibration type in comparison with whole-body vibration is recommended for ACL-reconstructed patients.

Vibration frequency higher than 100 Hz is preferred in ACL-reconstructed rehabilitation.

**Supplementary Information:**

The online version contains supplementary material available at 10.1186/s10195-021-00589-5.

## Introduction

Approximately 250,000 anterior cruciate ligament (ACL) injuries annually occur in the USA, and individuals with ACL injuries are three to five times more likely to develop knee osteoarthritis (OA) than healthy controls [1]. Moreover, the experience of posttraumatic OA contributes to a sedentary lifestyle with comorbidities such as cardiovascular diseases [2]. Importantly, in young adults in the productive years of their life within the workforce, this complication increases their economic burden beyond healthcare costs.

In injured ACL patients, increased hamstring activation for stabilization of joint through coactivation was measured, while in healthy controls quadriceps was the main muscle for joint stability [3, 4]. Quadriceps isokinetic strength deficits have been reported following ACL injury, reconstruction, and rehabilitation between 6 months and 15 years [5]. Postural control or balance has been closely attached to proprioception and neuromuscular control, though there exists some controversy on this subject. While some authors suggest that, in comparison with healthy subjects, ACL-injured patient’s greatest deficit lies in postural control during single-leg stance with both eyes closed [6, 7], other authors have reported no evidence of deficits in postural control among ACL-deficient and reconstructed-ACL patients [8, 9]. These phenomena have an important role in the design of ACL injury rehabilitation programs.

Hopkins and Ingersoll offered explanations for traditional rehabilitation programs’ shortcomings. They demonstrated that exercises for strengthening of quadriceps are ineffective at restoring muscle function in association with neural inhibition according to arthrogenic muscle inhibition (AMI) [10]. In fact, patients with neuromuscular deficiencies do not have the capacity to overload muscle fibers for proper force generation. Therefore, innovative rehabilitation methods are needed to fight AMI. Vibration therapy (VT) is a rehabilitation modality that has been reported to improve muscle function, even on the basis of electromyography studies [11, 12]. VT is defined as “a forced oscillation during which energy is transferred from an actuator (vibration device) to a resonator (human body)” [13]. VT effectiveness in neuromuscular control is mediated by additional mechanisms, including motor unit synchronization, central control, and intramuscular coordination [14–16]. Also, muscle temperature and blood flow enhancement are recorded using VT [17].

The aim of this systematic review and meta-analysis is to review and summarize all available evidence to assess the effectiveness of VT for quadriceps and hamstring strength, and postural stability, among ACL-reconstructed patients.

## Materials and methods

This systematic review and meta-analysis was conducted based on the Preferred Reporting Items for Systematic Reviews and Meta-Analyses (PRISMA) [18].

### Search strategy

The systematic search was conducted by two independent investigators using international electronic databases of PubMed, Scopus, Embase, Web of Science, EBSCO, Cochrane Central, and Google Scholar using a combination of these keywords based on the PICO of systematic review and meta-analysis. Search strategy in PubMed included: (“whole body vibration” OR “vibration therapy” OR mechanical vibration) AND (“anterior cruciate ligament reconstruction”) AND (“strength”, OR “postural stability” OR “neuromuscular control”). The bibliographical search was restricted to randomized controlled trials published prior to February 2020 with no restriction about language. Also, cross-referencing of relevant review articles was added. We contacted study authors via email to identify additional data for analysis.

### Inclusion criteria

Randomized clinical trials comparing exercise with and without VT were included. We assessed studies that were performed on adults (more than 18 years old), used VT for at least 1 week, and reported variables as mean differences and SDs both in the control and intervention groups. Additionally, studies with rehabilitation for the control group consisting of at least strengthening exercises (for quadriceps and hamstring) and proprioception/balance training were selected.

The outcome measures evaluated were quadriceps and hamstring peak torque, and postural stability, in adult individuals with ACL reconstruction. In the case of more than one article for a dataset, we chose the more complete one. Trials with more than two arms were considered as separate studies.

### Exclusion criteria

In the current review, we excluded in vitro studies; studies on animals; studies with a cohort, cross-sectional, and case–control design; review articles; and trials without a control group. Studies that did not include vibration therapy in their interventions were excluded. We excluded studies if the publication was in abstract form only. Studies lacking data that were necessary for meta-analysis (e.g., pre-intervention and post-intervention means and standard deviations) were also excluded.

### Data extraction

Two independent researchers (N.M. and S.P.) extracted the following data from eligible studies: name of the first author, publication year, individuals’ characteristics (mean age and sex), design, sample size (control and intervention groups), time interval from surgery to intervention, type of intervention (whole-body vibration or local vibration), VT parameters (frequency of vibration, amplitude, acceleration, and repetition/time), duration of intervention, and mean changes and SDs of outcome variables (quadriceps and hamstring peak torque, open-eye and closed-eye postural stability). When data for muscle torques were reported in different positions (angle), we used the most frequently used one.

### Quality assessment

Two independent reviewers (N.M. and M.K.) assessed the quality of the included studies using Jadad scoring (Oxford quality scoring system) for allocating the trials a score of between 0 (very poor) and 5 (rigorous) containing five questions [19]. Point 1 was for “positive” answers for each question and −1 for “negative.” If the response was “not described,” point 0 was allocated. The sum of all responses (whether positive or negative) gives the final score. The studies with a score equal and greater than 3 points were considered as high-quality studies.

Risk of bias was assessed using the Cochrane Collaboration’s tool with domains including performance bias, allocation concealment, detection bias, reporting bias, and attrition bias. The risk of bias was classified as low, unclear, and high for each domain [20].

Additionally, we evaluated the quality of each study’s intervention based on the criteria given by the International Society of Musculoskeletal and Neuronal Interactions (ISMNI) for reporting VT intervention studies, consisting of 13 factors about the WBV platform parameters and participants’ position during intervention [21].

### Statistical analysis

The means and related SDs of study outcomes before and after the intervention were used to calculate the standard mean differences using the fixed-effects model in meta-analysis. We used the *I*^2^ and Cochran’s *Q* test to assess the heterogeneity, and random-effects models were used if *I*^2^ value was higher than 70%, indicating high between-study heterogeneity [22]. Subgroup analysis and meta-regression analysis were used for assessing the potential source of heterogeneity. Moreover, sensitivity analysis was used for assessing the effect of every single study on the overall pooled estimate in meta-analysis. Egger test and visually checking the funnel plot were used for assessing publication bias. Stata software version 11 (Stata Corp, College Station, TX, USA) was used for all statistical analysis, and a *P*-value less than 0.05 was considered significant.

## Results

### Study selection, quality assessment, and characteristics

**Fig. 1:**
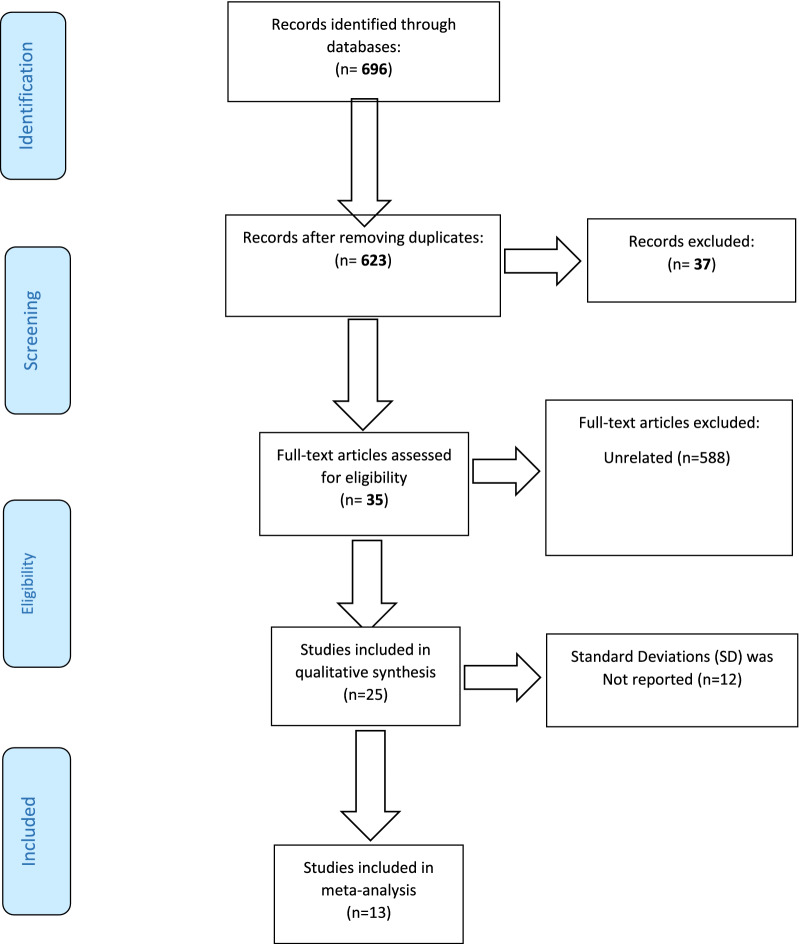
PRISMA flowchart

At the beginning of electronic database searching, we found 696 research studies. A total of 37 studies detected in more than one database and 588 other studies did not meet inclusion criteria. A total of 25 studies were assessed for eligibility. Twelve studies were eliminated because they did not report essential quantitative data, e.g., mean and its corresponding standard deviation (SD), or were congress abstracts or online abstracts studies for which we could not get the full text despite contacting their corresponding author for more information via email. Finally, we assessed 13 studies in the quality assessment stage to import for analysis [23–35] (Fig. 1). The mean score of the Jadad evaluation tool for methodological quality assessment was 3.75 out of 5. This means that most studies were high-quality studies, except for five [23, 24, 26, 32, 34]. The most common methodological weakness of studies was the lack of reports about withdrawals and blinding methods. All the trials adhered to randomization.

This meta-analysis evaluated a total of 407 patients. The sample size for each study ranged from 20 to 56. Overall, 175 (42.7%) of studied patients were women, with the mean age ranging from 20 to 31 years for all participants. Three studies used intervention 4 weeks after surgery, while most studies chose longer intervals ranging from 13 to 50 weeks. Characteristics of studies are presented in Table 1.

**Table 1:** Characteristics of included studies

Author, year	Study design	Study country	Groups	Number (% females)	Time interval from surgery to intervention	Graft type	Outcome measures	Duration	VT parameters
Salvarani, 2003	RCT	Italy	I: Conventional rehabilitation + WBV (*n* = 10) Control: Conventional rehabilitation (*n* = 10)	20 (85%)	1 month	Not presented	Mean of peak force (extension, flexion) EMG activity	2 weeks/10 sessions	Frequency: 30 Hz Amplitude: – Acceleration: – Repetition/time: 5/60 s
Brunetti, 2006	RCT	Italy	I: Conventional rehabilitation + LMV (*n* = 15) Control: Conventional rehabilitation (*n* = 15)	30 (61%)	1 month	Not presented	Extensor peak torque Postural control (open-eye and closed-eye) Ligament laxity EMG activity Knee ROM	1 week/3 sessions	Frequency: 100 Hz Amplitude: 5–15 µm Acceleration: – Repetition/time: 10/60 s
Fu, 2013	RCT	Hong Kong	I: Conventional rehabilitation + WBV (*n* = 19) Control: Conventional rehabilitation (*n* = 20)	48 (33%)	1 month	Hamstring graft	Joint position sense Postural control Knee ROM Ligament laxity Functional ability	8 weeks/16 sessions	Frequency: 20–60 Hz Amplitude: 2–4 mm Acceleration: – Repetition/time: –
Moezy, 2018	RCT	Iran	I: Conventional rehabilitation + WBV (*n* = 10) Control: Conventional rehabilitation (*n* = 10)	40 (50%)	3 months	Not presented	Joint position sense Postural control	12 weeks/12 sessions	Frequency: 30–50 Hz Amplitude: 2.5–5 mm Acceleration: – Repetition/time: 8–16/30-60 s
Berschin, 2014	RCT	Germany	I: Conventional rehabilitation + WBV (*n* = 20) Control: Conventional rehabilitation (*n* = 20)	40 (27%)	2 weeks	Patellar tendon graft	Extensor and flexor Peak torque Ligament laxity Postural control	10 weeks/– sessions	Frequency: 10–30 Hz Amplitude: 5–9 mm Acceleration: – Repetition/time: 2–6/60 s
Lee, 2015	RCT	USA	I: Conventional rehabilitation + WBV(*n* = 19)/LMV (n = 19) Control: Conventional rehabilitation (*n* = 18)	56 (50%)	–	Not presented	RTD Extensor peak torque	–/–	Frequency: 30 Hz Amplitude: – Acceleration: 2 g Repetition/time: 6/60 s
Costantino, 2017	RCT	Italy	I: Conventional rehabilitation + WBV (*n* = 19) Control: Conventional rehabilitation (*n* = 19)	38 (100%)	13 weeks	Not presented	Extensor and flexor Peak torque Extensor and flexor Maximum power	8 weeks/24 sessions	Frequency: 26 Hz Amplitude: 2.5 mm Acceleration: – Repetition/time:6/60 s
da Costa, 2017	RCT	Brazil	I: Conventional rehabilitation + WBV (*n* = 22) Control: Conventional rehabilitation (*n* = 22)	44 (0%)	14–18 weeks	Hamstring graft	Extensor peak torque EMG activity Postural control	–/–	Frequency: 50 Hz Amplitude: 4 mm Acceleration: – Repetition/time:10/30 s
Park, 2019	RCT	Korea	I: Conventional rehabilitation + LMV (*n* = 11) Control: Conventional rehabilitation (*n* = 13)	24 (40%)	1 week	Not presented	Extensor and flexor Peak torque Knee ROM	8 weeks/8 sessions	Frequency: 300 Hz Amplitude: – Acceleration: – Repetition/time:6–12/60 s
Pamukoff, 2016	RCT	USA	I: Conventional rehabilitation + WBV (*n* = 7)/LMV (*n* = 7) Control: Conventional rehabilitation (*n* = 6)	20 (60%)	Mean: 50 weeks	Patellar tendon/hamstring graft	Extensor peak torque Extensor AMT RTD MEP EMG activity	–/–	Frequency: 30 Hz Amplitude: – Acceleration: 2 g Repetition/time:6–6/60 s
Pamukoff, 2017	RCT	USA	I: Conventional rehabilitation + WBV (*n* = 7)/LMV (*n* = 7) Control: Conventional rehabilitation (*n* = 6)	20 (30%)	Mean:50 weeks	Patellar tendon/hamstring graft	RTD EMD Extensor peak torque	–/–	Frequency: 30 Hz Amplitude: – Acceleration: 2 g Repetition/time:6–6/60 s
Goetschius, 2019	RCT	USA	I: Conventional rehabilitation + WBV (*n* = 26) Control: Conventional rehabilitation (*n* = 21)	47 (–%)	Mean: 4.7 months	Patellar tendon/hamstring graft	Extensor peak torque MVIC	–/–	Frequency: 50 Hz Amplitude: 5 mm Acceleration: – Repetition/time:–
Bae, 2017	RCT	Korea	I: Conventional rehabilitation + WBV (*n* = 11) Control: Conventional rehabilitation (*n* = 11)	22 (10%)	–	Hamstring graft	Extensor peak torque Postural control	6 weeks/–	Frequency: 20–40 Hz Amplitude: – Acceleration: – Repetition/time: –

*AMT* active motor threshold, *RTD* rate of torque development, *MEP* motor evoked potential, *EMD* electromechanical delay, *MVIC* maximum voluntary isometric contraction, *EMG* electromyography, *RCT* randomized clinical trial, *LMV* local muscle vibration, *WBV* whole-body vibration

Considering the risk of bias assessment, excellent agreement was detected between evaluators (weighted Kappa 0.83). Table 2 presents details of the risk of bias assessment of the included studies. To summarize, the risk of bias was low in six studies [23, 24, 32–35], high in five studies [25, 28–31], and unclear in one study [26].

**Table 2:** Quality of each study’s intervention based on the criteria given by the International Society of Musculoskeletal and Neuronal Interactions (ISMNI) for WBV

Study	Q1	Q2	Q3	Q4	Q5	Q6	Q7	Q8	Q9	Q10	Q11	Q12	Q13	Score
Salvarani [23]	+	+	+	–	–	–	+	–	–	+	+	+	+	8
Fu [25]	+	+	+	+	–	–	+	+	–	+	+	+	+	10
Moezy [26]	–	+	+	+	–	–	+	+	–	+	+	+	+	9
Berschin [27]	+	+	+	+	–	–	+	+	–	+	+	+	+	10
Costantino [29]	+	+	+	+	–	–	+	+	–	+	+	+	+	10
da Costa [30]	+	+	+	+	–	–	+	–	–	+	+	+	+	9
Pamukoff [59]	+	+	+	–	+	–	+	–	–	+	+	+	–	8
Pamukoff [32]	+	+	+	+	+	–	+	–	–	+	+	+	–	8
Goetschius [34]	+	+	+	+	–	–	+	–	–	–	+	+	–	7
Bae [35]	+	+	+	–	–	–	+	+	–	–	+	+	–	7

Q1: brand and type, Q2: vibration type, Q3: vibration frequency, Q4: peak-to-peak displacement, Q5: peak acceleration, Q6: accuracy of parameters, Q7: skidding of the feet, Q8: vibration setting change, Q9: rationale for choosing, Q10: support devices, Q11: type of footwear, Q12: body position, Q13: exercise performed

The quality scores of each study based on the ISMNI recommendations are presented in Table 3. The mean score was 8.69 (ranging from 7 to 10) out of 13. All included studies have mentioned their used vibration device type and vibration frequency. None of the included studies has clearly discussed the accuracy of vibration parameters or rationale for choosing specific vibration settings.

**Table 3:** Risk of bias assessment of included studies

Study	Performance bias	Allocation concealment	Selection bias	Detection bias	Reporting bias	Attrition bias	
Salvarani [23]	Unclear	Unclear	No	Unclear	No	Unclear	2
Brunetti [24]	Unclear	Unclear	No	No	No	Unclear	3
Fu [25]	No	No	No	No	No	No	6
Moezy [26]	No	Unclear	No	Unclear	No	No	4
Lee [28]	No	No	No	No	No	No	6
Costantino [29]	No	No	No	No	No	No	6
da Costa [30]	No	No	No	No	No	No	6
Park [31]	No	Unclear	No	No	No	No	5
Pamukoff [59]	Unclear	Unclear	Unclear	No	No	Unclear	2
Pamukoff [32]	Unclear	Unclear	Unclear	No	Unclear	Unclear	1
Goetschius [34]	Unclear	Unclear	No	Unclear	No	Unclear	2
Bae [35]	Unclear	Unclear	No	No	No	Unclear	3

### Training protocol

The training protocols are presented in Table 1. All studies have declared frequency of vibration (10–300 Hz), while amplitude and acceleration were reported in seven and three studies, respectively. Duration ranged from 2 to 12 weeks, while five studies had a lack of data in this regard. The frequency of intervention use was different, ranging from two to eight times a week. All studies used VT in knee flexion position.

### Outcome measures

The outcome measures of interest were quadriceps/hamstring peak torque and postural control. All studies reported quadriceps peak torque, while three studies reported hamstring peak torque using the study protocol consisting of five repetitions with an angular speed of 90°/s and selection of the highest values. They registered Biodex System dynamometer software for measurement. Five studies reported postural control (open eye and closed eye) using the Biodex Stability System, which measures the deviation of the center of pressure from the center of gravity during stance. Postural stability was measured by anterior–posterior and medial–lateral stability indices, with a high score indicating poor balance.

Other outcomes, including joint position, ligament laxity, EMG activity, knee ROM, active motor threshold (AMT), rate of torque development (RTD), motor evoked potential (MEP), electromechanical delay (EMD), and maximum voluntary isometric contraction (MVIC), were introduced in evaluated studies with the limited number of studies making pooling impossible (Additional file 1: Fig. S2).

### Effectiveness of VT versus conventional rehabilitation for hamstring peak torque

The test for an overall effect of VT in comparison with conventional rehabilitation across the three trials was significant (*P* = 0.001), with an overall good effect size WMD of 12.67 (95% CI 4.51–20.83) that favored the VT compared with the control with *I*^2^ 64.8%, *P* = 0.058. Considering VT type, in the WBV subgroup, WMD decreased to 9.63 (95% CI 7.19–12.07), and in the LBV subgroup, WMD increased to 15.25 (95% CI 1.44–29.47) with decreasing heterogeneity (*I*^2^ 56.6%, *P* = 0.126). According to VT frequency, the subgroup with *F* < 50 Hz showed a WMD of 9.56 (95% CI 7.14–14.97), and the subgroup with *F* > 100 Hz had increased WMD of 20.84 (95% CI 11.75–29.93). There was no significant heterogeneity following subgroup analysis (*I*^2^ 0.0%, *P* = 0.698). After sensitivity analysis to assess the effects of each study, all three studies showed the same effect. Meta-regression confirmed that the year of study, vibration type, and vibration frequency were not sources of heterogeneity. Regarding Begg’s test and the symmetry of funnel plot (Additional file 2: Fig. S3), publication bias was not detected (*P* = 0.674) (Table 4).

**Table 4:** The pooled WMD of outcomes considering vibration frequency and vibration type as subgroups

Outcome	No. of included studies	Subgroup	Pooled WMD	95% CI	*I*^2^	*P* value for *I*^2^
ML postural control (CE)	3^26, 30, 35^	Total	–0.87	–1.48, –0.27	61.8	0.073
	2^26, 30^	< 50 Hz	–1.22	–1.94, –0.49	18.4	0.268
	NA 30	50–100 Hz	–0.52	–0.69, –0.35	–	–
AP postural control (CE)	4^26, 25, 35, 30^		–0.58	–2.78, 1.62	90.3	< 0.001
	3^26, 25, 30^	< 50 Hz	–1.63	–2.53, –0.72	27.4	0.254
	NA 30	50–100 Hz	2.82	1.37, 4.27	–	–
	3^26, 25, 30^	WBV	–0.49	–3.61, 2.62	93.5	0.088
	NA 35	LMV	–0.84	–2.25, 0.57	–	–
ML postural control (OE)	3^26, 25, 24^	Total	0.26	–1.26, 1.77	95.8	< 0.001
	2^26, 25^	< 50 Hz	–0.44	–0.78, –0.10	0.0	0.459
	NA 24	> 100 Hz	1.80	1.26, 2.34	–	–
	2^26, 25^	WBV	–0.44	–0.78, –0.10	0.0	0.459
	NA 24	LMV	1.80	1.26, 2.34	–	–
AP postural control (OE)	3^26, 25, 35^	Total	–0.49	–0.98, 0.01	1.0	0.364
	2^26, 25^	WBV	–0.53	–1.27, 0.21	50.5	0.155
	NA 30	LMV	–0.46	–2.24, 1.32	–	–
Hamstring PT	3^26, 35, 31^	Total	12.67	4.51, 20.83	64.8	0.058
	2^26, 35^	< 50 Hz	9.56	7.14, 14.97	0.0	0.698
	NA 31	> 100 Hz	20.84	11.75, 29.93	–	–
	NA 26	WBV	9.63	7.19, 12.07	–	–
	2^31, 35^	LMV	15.25	1.44, 29.47	56.6	0.129
Quadriceps PT	9^23, 24, 26, 28, 31, 32, 33, 34, 35^	Total	0.11	–0.06, 0.29	81.6	< 0.001
	5^26, 28, 31, 32, 35^	< 50 Hz	–0.30	–0.45, –0.17	0.0	0.648
	2^26, 34^	50–100 Hz	0.20	0.14, 0.26	0.0	0.582
	2^24, 32^	> 100 Hz	0.30	0.17, 0.44	0.0	0.889
	5^26, 28, 31, 32, 35^	WBV	0.09	–0.14, 0.32	58.9	0.032
	5^31, 32, 33, 34, 35^	LMV	0.16	–0.18, 0.49	89.4	< 0.001

*PT* peak torque, *AP* anterioposterior, *ML* mediolateral, *CE* closed-eye, *OE* open-eye, *NA* not applicable

### Effectiveness of VT versus conventional rehabilitation for quadriceps peak torque

This meta-analysis evaluated the nine trials to test the effect of VT in comparison with conventional rehabilitation on quadriceps peak torque among ACL-reconstructed patients. Total WMD was 0.11 (95% CI −0.06 to 0.29) with significant heterogeneity (*I*^2^ 81.6%, *P* < 0.001). Following subgroup analysis, the subgroup with vibration frequency > 100 Hz showed WMD of 0.30 (95% CI 0.17–0.44) compared with the control group, and heterogeneity improved to be insignificant (*I*^2^ 0.0%, *P* = 0.889). Sensitivity analysis showed elimination of the study by da Costa et al. caused WMD improvement to 0.15 (95% CI −0.06 to 0.29). Investigating sources of heterogeneity using meta-regression analysis showed that there was no source of heterogeneity considering the year of study, vibration type, and frequency. Based on Begg’s test and the status of the funnel plot (Additional file 2: Fig. S3), publication bias was not detected (*P* = 0.690).

### Effectiveness of VT versus conventional rehabilitation on postural control

Evaluating closed-eye postural control of ACL-reconstructed patients through four trials revealed the following: WMD of −0.87 (95% CI −1.48 to −0.27) with *I*^2^ 61.8%, *P* = 0.073 for mediolateral stability and WMD of −0.58 (95% CI −2.78 to 1.62) *I*^2^ 90.3%, *P* < 0.001 for anterioposterior stability, in favor of conventional rehabilitation. After subgroup analysis, although heterogeneity status improved (*I*^2^ 18.4%, *P* = 0.268 and *I*^2^ 27.4%, *P* = 0.254), WMD was again in favor of conventional rehabilitation, except for vibration frequency of 50–100 Hz with WMD of 2.82 (95% CI 1.37–4.27) but with only one study included in this subgroup (Table 5).

**Table 5:** Results of sensitivity analysis to assess the effects of each study on pooled WMD of outcomes

Outcome	No. of included studies		Pre-sensitivity analysis			Post-sensitivity analysis	
		Upper and lower of EF	95% CI	Pooled WMD	Excluded studies	95% CI	Pooled WMD
ML postural control (CE)	3^26, 25, 30^	Upper	–1.48, –0.27	–0.87	Brunetti	–1.27, –0.26	–0.87
		Lower			Fu	–2.18, 0.19	–0.99
AP postural control (CE)	4^26, 25, 35, 30^	Upper	–0.2.78, 1.62	–0.58	Moezy	–2.51, 2.69	0.09
		Lower			Salvarani	–2.77, 1.62	–0.57
ML postural control (OE)	3^26, 25, 24^	Upper	–1.26, 1.77	0.26	Brunetti	1.26, 2.33	1.80
		Lower			Moezy	–1.40, 0.04	–0.68
AP postural control (OE)	3^26, 25, 35^	Upper	–0.98, 0.01	–0.49	Salvarani	–0.98, 0.01	–0.48
		Lower			Fu	–1.60, –0.12	–0.86
Hamstring PT	3^26, 35, 31^	Upper	4.51, 20.83	12.67	All	4.51, 20.83	12.67
		Lower			All	4.51, 20.83	12.67
Quadriceps PT	9^23, 24, 26, 28,31, 32, 33, 34, 35^	Upper	–0.06, 0.29	0.11	da Costa	–0.01, 0.33	0.15
		Lower			Salvarani	–0.09, 0.26	0.08

*PT* peak torque, *AP* anterioposterior, *ML* mediolateral, *CE* closed-eye, *OE* open-eye

Open-eye evaluation of postural control of ACL-reconstructed patients revealed the following: WMD of 0.26 (95% CI −1.26 to 1.77) with *I*^2^ 95.8%, *P* < 0.001 for mediolateral stability, and WMD of −0.49 (95% CI −0.98 to 0.01) *I*^2^ 1.0%, *P* = 0.364 for anterioposterior stability. To summarize, only open-eye mediolateral postural control was improved in the VT group in comparison with the conventional group with improved effect in vibration frequency of > 100 Hz and LBV type (both WMDs 1.80, 95% CI 1.26–2.34) for a single study. After omitting the study by Brunetti et al., WMD increased to 1.80 (95% CI 1.26–2.33) in favor of VT. Heterogeneity changed to being insignificant after subgroup analysis (*I*^2^ 0.0%, *P* = 0.459). Meta-analysis was employed to determine the sources of heterogeneity. Evaluating closed-eye AP postural control, vibration frequency was found to be the source of heterogeneity [B (SE) 4.43 (0.18, 8.68), *P* = 0.046]. Considering Begg’s test and funnel plot (Additional file 2: Fig. S3), publication bias was not detected (*P* = 0.846) (Table 6).

**Table 6:** Univariate meta-regression analysis for assessing the effect of suspected variables on the pooled WMD of outcomes

Outcome	Variable	β	95% confidence interval for β	*P* value
ML postural control (CE)	Year of study	0.12	–0.59, 0.85	0.264
	Vibration type	–	–	–
	Vibration frequency	0.69	–5.02, 6.42	0.365
	Vibration time (week)^a^	0.03	–1.11, 1.17	0.811
AP postural control (CE)	Year of study	0.42	–0.71, 1.56	0.249
	Vibration type	0.34	–14.12, 14.81	0.927
	Vibration frequency	4.43	0.18, 8.68	**0.046**
	Vibration time (week)^a^	0.29	–4.41, 5.00	0.557
ML postural control (OE)	Year of study	–0.23	–3.86, 3.38	0.556
	Vibration type	–2.23	–6.37, 1.89	0.092
	Vibration frequency	1.11	–0.94, 3.18	0.092
	Vibration time (week)^a^	–0.17	–3.19, 2.84	0.599
AP postural control (OE)	Year of study	0.10	–1.02, 1.23	0.444
	Vibration type	–	–	–
	Vibration frequency	–0.06	–13.50, 13.36	0.958
	Vibration time (week)^a^	–	–	–
Hamstring PT	Year of study	5.64	–24.84, 36.12	0.256
	Vibration type	–5.62	–139.26, 128.01	0.678
	Vibration frequency	5.64	–24.84, 36.12	0.256
	Vibration time (week)^a^	–	–	–
Quadriceps PT	Year of study	–0.02	–0.06, 0.01	0.138
	Vibration type	–0.05	–0.50, 0.35	0.769
	Vibration frequency	–0.01	–0.28, 0.25	0.894
	Vibration time (week)^a^	–0.01	–0.02, 0.01	0.655

*PT* peak torque, *AP* anterioposterior, *ML* mediolateral, *CE* closed-eye, *OE* open-eye

^a^Time interval from surgery to intervention

## Discussion

The present review evaluated 13 RCTs including a total of 407 participants to examine the effectiveness of VT in the rehabilitation program of ACL-reconstructed patients in comparison with conventional rehabilitation.

Our pooled data indicated VT effectiveness in both hamstring and quadriceps muscles strengthening, with more prominent results for the hamstring. Impaired muscle activation following neuromuscular reorganization has been reported to contribute to strength loss and functional alteration. Most of the literature has focused on quadriceps and hamstring muscles [36-38]. Although previous rehabilitation protocols emphasized the role of the quadriceps, the most recent studies paid attention to the hamstring-to-quadriceps ratio for functional improvement after ACL injury [4, 39, 40]. The hamstring can limit anterior translation and rotation of the tibia on the femur, which compensates function of the ACL. Therefore, these two muscle imbalances lead to more functional disability following ACL injury and reconstruction [41]. Andrade and colleagues were the first to describe the arthrogenic muscle inhibition (AMI) phenomenon in which deformity of mechanoreceptors in an injured joint causes inhibition of the motor neurons of surrounding muscles by afferent signals alteration to the central nervous system (CNS) [42]. This process causes a defective cycle of more degenerative changes in an injured joint [10]. VT causes muscles and tendons to act like springs, storing energy slightly and releasing mechanical forces abruptly. An accumulation of mechanical energy within the body is of importance, which can harm muscles through increasing internal forces. Therefore, muscles can overcome damaging resonance via contractions for changes in stiffness and body position modifications [43]. VT acts through rapid alternating lengthening and shortening contractions, which triggers a tonic vibratory reflex [44]. This reflex coordinates agonist and antagonist muscles function in order to increase balance and movement impacting Golgi tendon organs [45]. Studies showed that the knee flexion position was efficient for absorption of energy by the lower extremity, and that the transmissibility of a signal reduced as the ankle, knee, and hip joint angles decreased [27, 46].

Moreover, VT has also been shown to improve balance and proprioception, which leads to a reduction in incidents of falling [47, 48]. Despite these desirable findings, there are also studies that show controversial results with regard to proprioception [49, 50], rate of torque development (RTD) [12], and muscle strength [51–53]. To explain the reason for discrepancies, we should pay attention to whether VTs were administered via different application methods (i.e., duration, amplitude, and frequency). In this study, just open-eye mediolateral postural control was improved by means of VT in comparison with conventional rehabilitation. It seems that VT does not have additional effects on proprioception and balance, and its observed effectiveness in postural control could be interpreted by muscle strengthening. Moreover, closed-eye postural control was better improved with conventional rehabilitation. Lee et al., through a study on the elderly population, suggested implementation of VT with closed-eye for more postural control [54].

We found better effectiveness of VT in hamstring/quadriceps strengthening in the local muscle vibration form. The majority of VT studies have focused on whole-body vibration (WBV) platforms, which are heavy and expensive devices. Local muscle vibration (LMV) may have similar effects and, furthermore, is accessible, portable, and cost-effective [12, 55]. There are just a few studies that compared LMV and WBV [28, 32, 55]. Concerning the evaluation of LMV effectiveness, applying the device on muscle–tendon, it is reported to be effective in improving leg extensor muscle strength [56], while other investigators reject it [57]. Although the direct application of LMV seems to be more effective than WBV affecting quadriceps through other muscles, the accumulative effect of WBV due to its probable additional proprioception improvements was questioned. However, our findings confirm the effectiveness of direct application.

Our findings showed better effectiveness with VT frequency higher than 100 Hz. Studies confirmed, within the broad range of frequencies available, that muscle relaxation occurs around 50 Hz, inhibition of spasticity at 100 Hz, pain relief at 200 Hz, and muscle training at 100–300 Hz. Although the exact mechanism is not introduced, studies suggested that vibration frequency can affect the excitement of the alpha motor neuron pool, which is involved in muscle strength and endurance [58].

### Study limitations and strengths

Strengths of this study include the use of formal approved International Society of Musculoskeletal and Neuronal Interactions (ISMNI) criteria, the Cochrane Collaboration’s tool to assess the risk of bias, and Jadad tool for methodology evaluation. The wide range of time intervals between surgery and intervention, the physical activity status of patients, and some missing data on vibration protocol in some studies are limitations of this review. Therefore, the present study did not recommend optimal complete vibration parameters owing to the lack of data, although it included some about vibration frequency and vibration type. Five out of the 13 studies included low-quality studies. Furthermore, in this review, no study assessed the isolated effect of WBV on outcome, and effectiveness evaluation was concomitant with conventional exercises. Vibration platform-related data as an effective variable on outcomes were not capable of pooling. Furthermore, long-term follow-up was seen in limited studies. Although graft type is of importance in the rehabilitation of patients after ACL reconstruction, most studies did not report the type of graft they used. Additionally, in some studies, graft type was declared but subgroup analysis based on graft type was not seen. Therefore, attention to graft type in studies on rehabilitation of ACL-reconstructed patients is recommended for future investigations.

## Conclusion

This study indicates the effectiveness of VT, especially LMV type with vibration frequency higher than 100 Hz, for rehabilitation of ACL-reconstructed patients. Although improvement in peak torque of hamstring and quadriceps muscles was seen, there was no significant improvement in postural control, especially closed-eye, in comparison with conventional rehabilitation.

## Supplementary Information

**Additional file 1.** Forest plot of quadriceps peak torque improvement following VT (A: Total, B: based on vibration frequency, C: based on vibration type).

**Additional file 2.** Funnel plot (A: Closed-eye medio-lateral postural control, B: Closed-eye anterio-posterior postural control, C: Open-eye medio-lateral postural control, D: Open-eye anterio-posterior postural control, E: Hamstring peak torque, F: Quadriceps peak torque).

## Data Availability

All data are presented in Additional files figures. In the case of special need, data will be available by contacting corresponding author.

## Abbreviations

VT: Vibration therapy
WBV: Whole-body vibration
LWV: Local muscle vibration
PTOA: Posttraumatic osteoarthritis
RCTs: Randomized clinical trials
ISMNI: International Society of Musculoskeletal and Neuronal Interactions
AMT: Active motor threshold
RTD: Rate of torque development
MEP: Motor evoked potential
EMD: Electromechanical delay
MVIC: Maximum voluntary isometric contraction
ACL: Anterior cruciate tendon
WMD: Weighted mean difference
AMI: Arthrogenic muscle inhibition
