# Supplementary material for: The effectiveness of vibration therapy for muscle peak torque and postural control in individuals with anterior cruciate ligament reconstruction: a systematic review and meta-analysis of clinical trials
Source: J Orthop Traumatol. 2021 Jul 14;22:28. doi: 10.1186/s10195-021-00589-5 (PMC8280257; doi:10.1186/s10195-021-00589-5)
Supplement: Supplementary file 2 — Additional file 2. Funnel plot (A: Closed-eye medio-lateral postural control, B: Closed-eye anterio-posterior postural control, C: Open-eye medio-lateral postural control, D: Open-eye anterio-posterior postural control, E: Hamstring peak torque, F: Quadriceps peak torque). [file 10195_2021_589_MOESM2_ESM.docx]

 A B

 C D

E F
